# Association of a homozygous *GCK* missense mutation with mild diabetes

**DOI:** 10.1002/mgg3.728

**Published:** 2019-06-14

**Authors:** Antonella Marucci, Tommaso Biagini, Rosa Di Paola, Claudia Menzaghi, Grazia Fini, Stefano Castellana, Giuliana Marcella Cardinale, Tommaso Mazza, Vincenzo Trischitta

**Affiliations:** ^1^ Research Unit of Diabetes and Endocrine Diseases Fondazione IRCCS Casa Sollievo della Sofferenza San Giovanni Rotondo (FG) Italy; ^2^ Unit of Bioinformatics Fondazione IRCCS Casa Sollievo della Sofferenza San Giovanni Rotondo (FG) Italy; ^3^ Pediatric Division Hospital F. Ferrari Casarano Italy; ^4^ Department of Experimental Medicine Sapienza University Rome Italy

**Keywords:** in‐silico analyses, monogenic diabetes, protein stability

## Abstract

**Background:**

Homozygous inactivating *GCK* mutations have been repeatedly reported to cause severe hyperglycemia, presenting as permanent neonatal diabetes mellitus (PNDM). Conversely, only two cases of *GCK* homozygous mutations causing mild hyperglycemia have been so far described. We here report a novel *GCK* mutation (c.1116G>C, p.E372D), in a family with one homozygous member showing mild hyperglycemia.

**Methods:**

*GCK* mutational screening was carried out by Sanger sequencing. Computational analyses to investigate pathogenicity and molecular dynamics (MD) were performed for GCK‐E372D and for previously described homozygous mutations associated with mild (*n* = 2) or severe (*n* = 1) hyperglycemia, used as references.

**Results:**

Of four mildly hyperglycemic family‐members, three were heterozygous and one, diagnosed in the adulthood, was homozygous for GCK‐E372D. Two nondiabetic family members carried no mutations. Fasting glucose (*p* = 0.016) and HbA1c (*p* = 0.035) correlated with the number of mutated alleles (0–2).

In‐silico predicted pathogenicity was not correlated with the four mutations’ severity. At MD, GCK‐E372D conferred protein structure flexibility intermediate between mild and severe *GCK* mutations.

**Conclusions:**

We present the third case of homozygous *GCK* mutations associated with mild hyperglycemia, rather than PNDM. Our in‐silico analyses support previous evidences suggesting that protein stability plays a role in determining clinical severity of *GCK* mutations.

## 1 BACKGROUND

Glucokinase (GCK, OMIM 138079) catalyzes the phosphorylation of glucose to glucose‐6‐phosphate, the first step in glucose metabolism. In pancreatic beta cells, GCK plays a role on insulin secretion acting as a sensor of extracellular glucose concentration; accordingly, altered GCK function may affect glucose homeostasis causing either hyperglycemia or hypoglycemia, depending on whether the enzymatic activity is reduced or increased, respectively (Osbak et al., 2009).

It is well established that heterozygous loss of function *GCK* mutations cause maturity‐onset diabetes of the young (MODY) also known as MODY2 (*GCK*‐MODY; OMIM entry #125851). This subtype of monogenic diabetes is characterized by mild fasting hyperglycemia, with patients usually needing no pharmacological treatment and having a very low risk of chronic diabetic complications (Steele et al., 2014).

Conversely, homozygous inactivating mutations of *GCK* have been repeatedly reported (Bennett et al., 2011; Njølstad et al., 2001; Raimondo et al., 2014; Turkkahraman et al., 2008) to cause permanent neonatal diabetes mellitus (PNDM; OMIM entry #606176). Unlike *GCK*‐related‐MODY, *GCK*‐related‐PNDM is characterized by severe hyperglycemia.

This scenario was partly questioned by a recent study (Raimondo et al., 2014) reporting in white French Canadians two cases of homozygous *GCK* mutations causing a mild, childhood‐onset diabetes, rather than a severe, neonatal‐onset disease. More generally, this study was instrumental in highlighting that, according to their ability to affect protein stability, homozygous *GCK* mutations cause a spectrum of glucose homeostasis abnormalities, ranging from very mild to severe hyperglycemia.

We here describe a new homozygous *GCK* missense mutation (c.1116G>C, p.E372D) causing in a white Italian woman, a mild form of diabetes diagnosed at 22 years of age, during pregnancy. To the best of our knowledge, this is the third case of homozygous *GCK* mutation associated to diabetes occurring outside infancy.

## 2 METHODS

### 2.1 Sequencing studies

Genomic DNA was extracted and *GCK* (RefSeq NM_000162.3) studied by direct Sanger sequencing and by a next generation sequencing (NGS) guided approach, as described in Data S1.

### 2.2 In‐silico studies

#### 2.2.1 Pathogenicity prediction

The impact of the four variants on GCK was assessed by sixteen pathogenicity prediction software packages. See Data S1.

#### 2.2.2 Molecular dynamics simulation

The atomic model of the wild‐type GCK protein (GCK‐WT) in complex with a molecule of glucose was obtained from the Protein Data Bank (id:1V4S). GCK‐WT was mutated in‐silico through UCSC Chimera to introduce the variations of interest. The resulting models were subjected to Molecular Dynamics (MD) simulation, as previously described (Biagini et al., 2017) and reported in Data S1.

### 2.3 Statistical analyses

Data on correlation between glucose or HbA1c levels and number of mutated alleles were evaluated by linear regression model and reported as Beta (β) value ± Standard Error (SE). A two sided *p*‐values < 0.05 were considered as statistically significant. Statistical analyses were performed by using R, version 3.3.3 (Team & RDC, 2017).

## 3 RESULTS

### 3.1 Clinical data

A genetic counseling was asked for a 3‐years‐old boy, subject IV‐1 (family's pedigree is shown in Figure S1), whose blood glucose concentrations were 89 mg/dl and 213 mg/dl at fasting and 2 hr after an oral glucose load, respectively. He also had a prediabetes HbA1c level of 44 mmol/mol (6.2%). Tests for antibodies against insulin, glutamic acid decarboxylase, and protein tyrosine phosphatase–like molecule IA‐2 were negative. Subject III‐2 (30‐years‐old) showed impaired fasting glucose (114 mg/dl) and a prediabetes HbA1c level of 41 mmol/mol (5.9%) while in subject II‐3 (51‐years‐old), diabetes was diagnosed at 22 years during her first pregnancy. Since then, she was treated with metformin (discontinued at our Institution after genetic diagnosis was made). Her recent fasting glucose and HbA1c levels ranged 129–140 mg/dl and 44–54 mmol/mol (6.2%–7.1%), respectively. Finally, in subject II‐2 (61‐years‐old) diabetes was diagnosed at 50 years; under continuous metformin treatment (as before, this was discontinued after genetic diagnosis), last fasting blood glucose concentration was 94 mg/dl while HbA1c level was 39 mmol/mol (5.7%), the prediabetes threshold. In addition, subjects III‐3 and IV‐2 showed normal fasting glucose levels, equal to 97 mg/dl and 87 mg/dl, respectively. HbA1c level in subject III‐3 was 37 mmol/mol (5.5%), while no HbA1c level was available for subject IV‐2. At Sanger sequencing, proband resulted heterozygous for a new *GCK* missense mutation c.1116G>C, p.E372D. No additional mutations were found in any of the additional 27 monogenic diabetes genes screened by custom targeted NGS panel. The same mutation was screened by Sanger sequencing in all family members. Quite surprisingly, while subjects III‐2 and II‐2 were heterozygous, subject II‐3, the proband's paternal grandmother, turned out to be homozygous; no mutation was found in subject III‐3 and IV‐2. No information on consanguinity between subjects I‐1 and I‐2 was available. Table 1 shows all these measurements as well as additional clinical features of the four affected members.

**Table 1:** Clinical features of family members carrying the GCK (c.1116G<C, p.E372D) mutation

	Subject IV−1	Subject III−2	Subject II−3	Subject II−2
Gender (M/F)	M	M	F	F
Age at diagnosis of hyperglycemia (years)	3	30	22	50
BMI (kg/m^2^)	17.6	27.5	27.2	32.1
Fasting glucose (mg/dl)	86–89	114	129–140	96–94
HbA1c (mmol/mol)	44	41	44–54	39

Abbreviations: F, Female; M, Male.

Of interest, a clear correlation was observed between both fasting glucose (*β* ± SE = 21.2 ± 6.7 mg/dl, *p* = 0.016) and HbA1c (*β* ± SE = 0.8 ± 0.2%, *p* = 0.035) levels and the number of mutated alleles (0–2) across the six family study members (Figure S2, panels a and b).

### 3.2 In‐silico analyses

Our GCK‐E372D variant was analyzed in‐silico and compared to previously described *GCK* homozygous mutations that were associated to a wide range of clinical severity and protein stability. In detail, the GCK‐H50D mutation, reported to dramatically hamper protein stability and to cause a severe form of PNMD, was chosen as a reference severe mutation (Raimondo et al., 2014), while GCK‐D160N and GCK‐V226M, carried by mild hyperglycemic subjects and with mild impact on protein stability, were chosen as reference mild mutations (Raimondo et al., 2014).

#### 3.2.1 Pathogenicity prediction

GCK‐E372D was considered harmful by 11 out of 16 predictor tools, while GCK‐H50D, GCK‐D160N, and GCK‐V226M were predicted harmful by 14, 14, and 15 predictors (Table S1), respectively. Clearly, no parallelism was observed between predicted pathogenicity and clinical severity.

#### 3.2.2 MD simulation

The GCK molecule contains a small and a large domain, enveloping the glucose‐binding site. Amino acid residues 1–64 and 206–439 belong to the large domain, while amino acid residues 72–201 and 445–465 belong to the small one. Amino acid residues 65–71, 202–205 and 440–444 form three loops connecting these two domains (Kamata, Mitsuya, Nishimura, Eiki, & Nagata, 2004).

**Figure 1:**
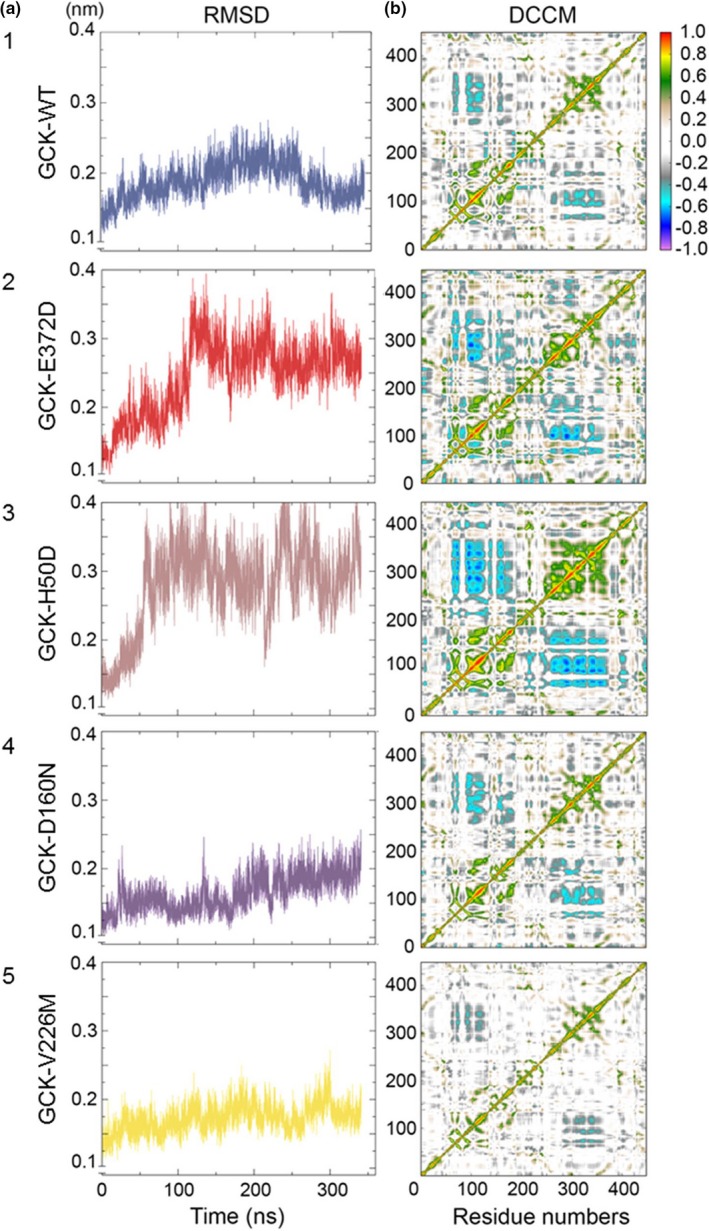
Molecular dynamics analysis. Panel a shows the backbone Root‐Mean‐Square‐Deviation (RMSD) of the native and mutant structures of the GCK protein. The blue, red, brown, violet, and yellow lines indicate the native GCK‐WT, the GCK‐E372D, the GCK‐H50D, the GCK‐D160N, and the GCK‐V226M mutant structures, respectively. Panel b shows the Dynamic‐Cross‐Correlation‐Maps (DCCMs); each matrix displays the long‐range interactions between the atoms forming the small and the large domain of GCK native and mutant proteins. Red to green peaks in the maps are indicative of strong to moderate positive correlation, dark to light blue peaks are indicative of strong to moderate anticorrelation between the indicated residue numbers

The dynamics of GCK upon glucose binding, with the protein switching from an inactive (open) to an active (close) conformation (Kamata et al., 2004), was assessed in terms of Root‐Mean‐Square‐Deviation (RMSD, Data S1). Compared to GCK‐WT (Figure 1a, panel‐1), our novel GCK‐E372D mutation (Figure 1a, panel‐2) showed higher RMSD deviations; an even greater difference toward higher RMSD deviations was shown by the severe, PNMD‐related GCK‐H50D mutation (Figure 1a, panel‐3). Conversely, the two mild, GCK‐D160N and GCK‐V226M mutations (Figure 1a, panel‐4 and panel‐5) showed lower RMSD deviation, as compared to GCK‐WT. Mutual atomic motions were represented by Dynamic‐Cross‐Correlation‐Maps (DCCMs, Data S1). In GCK‐WT (Figure 1b, panel‐1), atoms in the small domain moved in an anticorrelated way with those in the large domain. Such anticorrelation was clearly increased in GCK‐E372D (Figure 1b, panel‐2) and even more in GCK‐H50D (Figure 1b, panel‐3), while it was conserved, at least partly in GCK‐D160N and GCK‐V226M (Figure 1b, panel‐4 and panel‐5).

**Figure 2:**
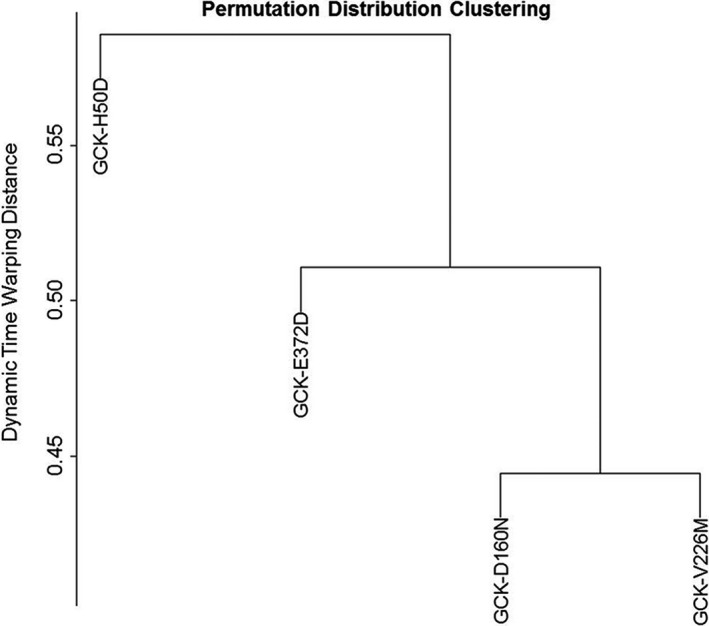
Permutation Distribution Clustering. Clustering of the binding pocket dynamics of GCK native and mutant proteins over time. Similarities, that is, distances between time series were evaluated by Dynamic Time Warping (DTW)

Finally, hierarchical clustering analysis of the binding pocket volume dynamics over time showed that, as compared to GCK‐WT, GCK‐E372D lies in between the two mild GCK‐D160N and GCK‐V226M and the severe, PNMD‐related GCK‐H50D mutations (Figure 2).

## 4 DISCUSSION

Homozygous inactivating mutations of *GCK* have been repeatedly reported to contribute to PNDM, with only two exceptions, described in 9 and 15 years old white French Canadians showing mild hyperglycemia totally superimposable to that observed in GCK‐related MODY conditions (Raimondo et al., 2014). We here describe a novel *GCK* missense mutation p.E372D that in an Italian family co‐segregates with mild impairment of glucose homeostasis not only in heterozygous patients but also in the only homozygous family member who was diagnosed as diabetic in the adult life. Fasting glucose was below the threshold of diabetes diagnosis in all three heterozygous individuals and within the normal range in two of them. In addition, HbA1c level was in the range of prediabetes (ADA, 2019) in all of them. These results are fully compatible with previous reports in patients with *GCK*‐MODY (Chakera et al., 2015; McDonald & Ellard, 2015), although they may have also been partly influenced by the ongoing treatment with metformin that two of our cases were taking when referred to our Institute. It is worth noticing that in the proband, glucose level at 120 min at OGTT had a delta increase as compared to fasting level of 124 mg/dl that is much higher than what usually reported for *GCK*‐MODY patients. Interestingly, among our family members, a clear correlation between the number of mutated alleles and both fasting glucose and HbA1c levels was observed (i.e., gene‐dose effect). To the best of our knowledge, this is the third example of *GCK* homozygous missense mutation, which, rather than causing PNDM, causes a typical *GCK*‐related mild hyperglycemia.

To address the biological significance of our new mutation and to compare it to previously reported *GCK* mutations showing a wide range of clinical severity and dysfunctional biological features (Raimondo et al., 2014), in‐silico analyses were carried out.

All GCK mutations analyzed by a composite score, comprising 16 distinct pathogenicity tools (Table S1), were predicted harmful, with no parallelism with disease severity.

Dynamics of GCK, carried out under conditions simulating glucose binding, suggest that GCK‐E372D confers a more flexible structure as compared to GCK‐WT. Notably the severe, PNDM associated GCK‐H50D mutation, showed effects that, though toward the same directions, were much stronger than those of GCK‐E372D. Conversely, both mild GCK‐D160N and GCK‐V226M mutations induced opposite effects suggesting a less flexible, more stable GCK protein than GCK‐WT. In all, our in‐silico MD analyses, including also mutual atomic motions and hierarchical clustering analysis indicate that the novel GCK‐E372D mutation lies functionally somewhere in between the mild GCK‐D160N and GCK‐V226M and the severe PNDM‐related GCK‐H50D mutations.

Previous in vitro experimental evidences suggested that the degree of GCK clinical severity is inversely related to protein stability (Raimondo et al., 2014). In fact, our present in‐silico data are fully compatible with the previously reported relative differences in protein stability among severe and mild GCK mutations (Raimondo et al., 2014). Within this frame, however, the increased flexibility showed by our novel GCK‐E372D mild mutation as compared to GCK‐WT suggests that mild GCK mutations, though more stable than severe mutations, are not necessarily characterized by absolutely increased protein stability.

In conclusion, we here report the third evidence of a *GCK* homozygous mutation that, rather than causing PNDM, causes mild abnormalities of glucose homeostasis diagnosed out of infancy. Our present data reinforce the concept of genetic heterogeneity in the subset of GCK‐related diabetes that may translate into a wide range of phenotypes, from the more extreme to intermediate ones. In‐silico analyses together with previous in vitro experimental data (Raimondo et al., 2014), support the idea that protein stability plays a role on mutation clinical severity.

## CONFLICT OF INTEREST

The authors declare no conflict of interest.
