# Supplementary material for: Association of a homozygous GCK missense mutation with mild diabetes
Source: Mol Genet Genomic Med. 2019 Jun 14;7(7):e00728. doi: 10.1002/mgg3.728 (PMC6625123; doi:10.1002/mgg3.728)
Supplement: Supplementary file 1 [file MGG3-7-e00728-s001.docx]

**Supplementary Methods**

**Sequencing studies**

After obtaining informed consent for the GCK gene mutational analysis from all family members, genomic DNA was extracted from EDTA whole-blood by using QIAmp DNA Blood Kit (Qiagen Inc., Valencia, CA). Exons, their flanking introns (20bp), and promoter region of the *GCK* (RefSeq NM_000162.3) were amplified by polymerase chain reaction (PCR) using gene-specific oligonucleotide primers. All amplicons were sequenced using a BigDye Seq kit 1.1 (Applied Biosystems, Foster City, CA), according to the manufacturer’s instructions on ABI 3130 DNA sequencer (Applied Biosystems, Foster City, CA). The results were analyzed with Sequencher software v5.4.6 (GeneCodes, Ann Arbor, MI) and Alamut Visual 2.10 Interactive Biosoftware. All mutations were validated at least once on a second PCR product.

Proband’s DNA was subjected to NGS analysis of the following monogenic diabetes genes (i.e. *ABCC8, APPL1, BLK, CEL, GATA4, GATA6, GCK, HNF1A, HNF1B, HNF4A, PDX1, KCNJ11, KLF11, NEUROD1, PAX4, RFX6, TRMT10A, WFS1, ZFP57, EIF2AK3, FOXP3, GLIS3, IER3IP1, NEUROG3, PTF1A, SLC19A2, SLC2A2*) were also sequenced by means of custom target enrichment KAPA HyperPlus Library Preparation Kit and Nimblegen SeqCap EZ kit (Roche, Pleasanton, CA), according to the manufacturer’s instruction on NextSeq 500 (Illumina Inc., San Diego, CA).

**In-silico studies**

*Pathogenicity prediction*

The impact of the four variants p.H50D, p.D160N, p.V226M and p.E372D on GCK was assessed by an approach extending a similar one we recently developed (Pezzilli et al., 2018) and that comprises sixteen pathogenicity prediction software packages (Supplementary Table S1). These were chosen because of their maintenance frequency, estimation congruency or superior classification records (Castellana, Fusilli, & Mazza, 2016; Castellana & Mazza, 2013). Their results were binarized to 1, when the following conditions were met, or to 0, otherwise: SIFT score<0.05, PolyPhen2 HDIV>0.453, fathmm<0, fathmm-MKL>0.5, MetaLR>0.5, MetaSVM>0, DANN>0.8, VEST3>0.75, CADD>20, PROVEAN<-2.5, MutationAssessor>1.9, Eigen>0.29, GenoCanyon≥0.49, M-CAP=D, LRT=D and MutationTaster=A or D. It should be noted that M-CAP, LRT and MutationTaster provide categorical classifications only, which are D, N for M-CAP, where D stands for “deleterious” and N for “predicted neutral”; D, N and U for LRT, where U stands for “unknown”; A, D, N, P for MutationTaster, where A stands for “disease causing automatic”, namely predicted as disease causing in ClinVar, and P for “polymorphism-automatic”, namely predicted as neutral in ClinVar. Finally, an agglomerative pathogenicity score was obtained for each variant by summing the 16 binary scores as previously derived. The variants were considered pathogenic by majority vote, namely if their total pathogenicity scores were >8.

*Molecular dynamics*

An in-depth analysis was further conducted on the 3D atomic model of GCK, in complex with a molecule of glucose, which was obtained from the Protein Data Bank (PDB id: 1V4S). The residues of the considered model correspond to the actual residues 14-465 of the GCK protein. The wild-type GCK protein was mutated in-silico through UCSC Chimera to introduce any variation of interest. The resulting models were preprocessed and prepared for Molecular Dynamics (MD) simulation, as previously described (Biagini et al., 2017). Gromacs tools were used to calculate the (i) Root-Mean-Square-Deviation (RMSD), which measures the average distance between all heavy atoms (in this case Cα atomic coordinates) with respect to the X-ray structure; (ii) Dynamic-Cross-Correlation-Maps (DCCMs), which allows investigating the long-range interactions of atoms; peaks, corresponding to the Cij elements of the map, are indicative of strong to moderate positive correlation (red to green), or of strong to moderate anti-correlation (dark to light blue) between residues i and j. The data obtained were then analyzed by Dynamic Time Warping Distance (DTW) with the dtw R package to evaluate similarities between volume profiles during simulation. Permutation distribution clustering with the complete method was applied to the computed DTW distances. Comprehensive measures of similarity and hierarchical clustering were computed with the pdc R package (ver. 3.4.2).

**Bibliography**

Biagini, T., Chillemi, G., Mazzoccoli, G., Grottesi, A., Fusilli, C., Capocefalo, D., . . . Mazza, T. (2017). Molecular dynamics recipes for genome research. Briefings in bioinformatics, bbx006. doi: 10.1093/bib/bbx006

Castellana, S., Fusilli, C., & Mazza, T. (2016). A broad overview of computational methods for predicting the pathophysiological effects of non-synonymous variants. Methods in Molecular Biology 1415, 423-440.

Castellana, S., & Mazza, T. (2013). Congruency in the prediction of pathogenic missense mutations: state-of-the-art web-based tools. Briefings in bioinformatics, 14(4), 448-459. doi: 10.1093/bib/bbt013

Pezzilli, S., Ludovico, O. , Biagini, T., Mercuri, L. , Alberico, F., Lauricella, E., . . . Prudente, S. (2018). Insights from molecular characterization of adult patients of families with multigenerational diabetes. Diabetes 67, 137–145.

**
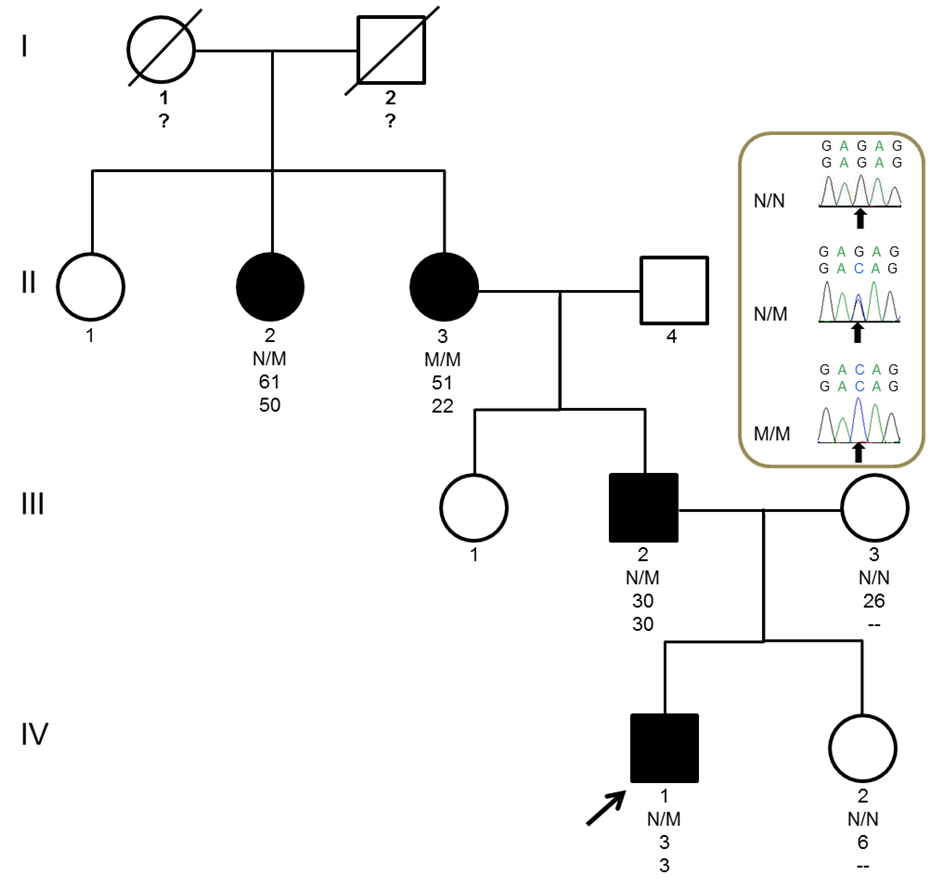
**

**Supplementary Figure S1**

**Family’s Pedigree**

Subjects in each generation were numbered. Round and square symbols denote females and males, respectively. Black-filled symbols denote individuals with diabetes or with impaired fasting glucose (IFG).

The proband is indicated by an arrow. N/M and M/M denote presence of heterozygous or homozygous *GCK* (c.1116G>C, p.E372D) mutation, respectively; N/N indicates absence of mutation. For each individual, age at examination is reported under the corresponding symbol; for diabetic or IFG individuals age at diagnosis is indicated under age at examination.

Representative electropherograms of *GCK* (c.1116G>C) mutation are shown into the inset.


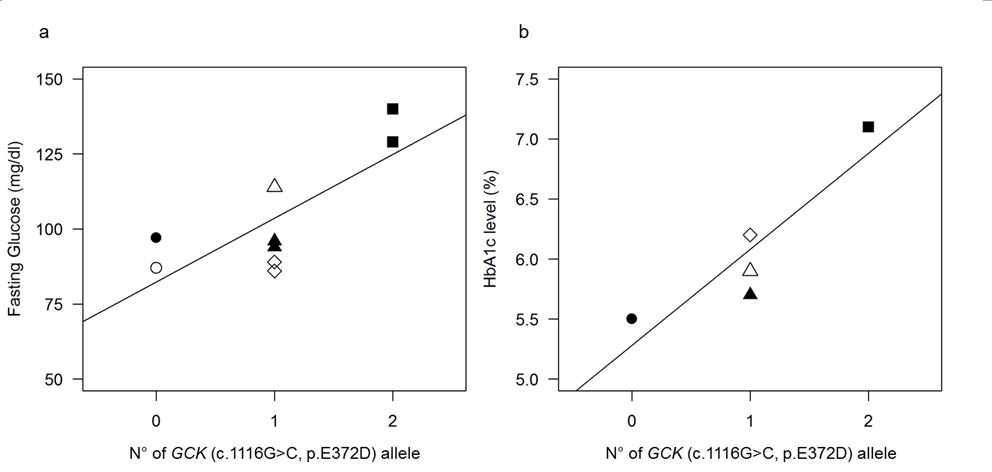


**Supplementary Figure S2**

**Correlation between number of mutated alleles and both fasting glucose and HbA1c levels**

Correlation between both fasting glucose (panel a) or HbA1c levels (panel b) and increasing number of *GCK* (c.1116G>C, p.E372D) mutated alleles across the 6 family study members were reported (i.e. 0 = no mutated alleles, 1 = one mutated allele, 2 = two mutated alleles). Different symbols indicate different family members as it follows: III-3 black circle, IV-2 white circle, III-2 white triangle, IV-1 black triangle, II-2 white rhombus and II-3 black square. Of note, for some subjects more than one fasting glucose measure were available and showed in the plot.

For each additional mutated allele (i.e. from 0 to 2) an increment of 21.25±6.72 mg/dl (p=0.016), or of 0.8±0.22 % (p=0.035) for fasting glucose and HbA1c levels, respectively, was observed.

**Supplementary Table S1. Pathogenicity prediction tools**

| **Pathogenicity**  **prediction tools** | **Threshold score** | **GCK-E372D** | | **GCK-H50D** | | **GCK-D160N** | | **GCK-V226M** | |
| --- | --- | --- | --- | --- | --- | --- | --- | --- | --- |
|  |  | ***Score*** | ***Prediction*** | ***Score*** | ***Prediction*** | ***Score*** | ***Prediction*** | ***Score*** | ***Prediction*** |
| SIFT | <0.05 | 0.153 | Tolerated | 0.015 | Deleterious | 0.034 | Deleterious | 0.001 | Deleterious |
| Polyphen2_HDIV | >0.453 | 0.994 | Probably damaging | 0.947 | Possibly damaging | 1.000 | Probably damaging | 1.000 | Probably damaging |
| Mutation Assessor | >1.9 | 1.690 | L=low | 1.705 | L=low | 0.715 | Neutral | 3.410 | M=medium |
| FATHMM | <0 | -4.130 | Deleterious | -5.240 | Deleterious | -4.770 | Deleterious | -4.860 | Deleterious |
| PROVEAN | <-2.5 | -0.700 | Neutral | -3.010 | Deleterious | -4.620 | Deleterious | -2.400 | Neutral |
| VEST3 | >0.75 | 0.747 | Neutral | 0.784 | Deleterious | 0.130 | Neutral | 0.972 | Deleterious |
| MetaSVM | >0 | 0.869 | Deleterious | 0.871 | Deleterious | 0.997 | Deleterious | 1.099 | Deleterious |
| MetaLR | >0.5 | 0.867 | Deleterious | 0.895 | Deleterious | 0.930 | Deleterious | 0.968 | Deleterious |
| CADD | >20 | 23.900 | Deleterious | 26.200 | Deleterious | 27.300 | Deleterious | 34.000 | Deleterious |
| DANN | >0.8 | 0.986 | Deleterious | 0.993 | Deleterious | 0.999 | Deleterious | 0.999 | Deleterious |
| fathmm-MKL coding | >0.5 | 0.852 | Deleterious | 0.975 | Deleterious | 0.991 | Deleterious | 0.940 | Deleterious |
| Eigen | >0.29 | 0.034 | Neutral | 0.398 | Deleterious | 0,466 | Deleterious | 0.889 | Deleterious |
| Geno Canyon | ≥0.49 | 1.000 | Deleterious | 1.000 | Deleterious | 1.000 | Deleterious | 1.000 | Deleterious |
|  | **Categorical classification** |  | | | | | | | |
| LRT | D, N, U | D | Deleterious | N | Neutral | D | Deleterious | D | Deleterious |
| Mutation Taster | A, D, N, P | D | Deleterious | D | Deleterious | D | Deleterious | A | Disease causing automatic |
| M-CAP | D, N | D | Deleterious | D | Deleterious | D | Deleterious | D | Deleterious |
| **Harmful/total prediction tools** | | **11/16** | | **14/16** | | **14/16** | | **15/16** | |

Pathogenicity prediction tools with threshold score or categorical classification. Reported categorical classification stand for: A=Disease causing automatic, D=Deleterious, N=Neutral, P=Polymorphism-automatic and U=Unknown.
